# Tertiary lymphoid structures in cancer: their development, composition, clinical prognosis, and the drivers of the anti-tumor responses

**DOI:** 10.1186/s43556-026-00562-w

**Published:** 2026-09-04

**Authors:** Zhi-chao Dou, Yu-chao Shi, Kun Meng, Jia-Rong Wang, Xin-hua Liang, Ya-Ling Tang

**Affiliations:** 1 State Key Laboratory of Oral Diseases & National Clinical Research Center for Oral Diseases &, Department of Oral Pathology, West China Hospital of Stomatology, Sichuan University, Chengdu, Sichuan China; 2 State Key Laboratory of Oral Diseases & National Clinical Research Center for Oral Diseases &, Department of Oral Medical Imaging, West China Hospital of Stomatology, Sichuan University, Chengdu, Sichuan China; 3 Department of Stomatology, The General Hospital of Western Theater Command PLA, Chengdu, China; 4 State Key Laboratory of Oral Diseases & National Clinical Research Center for Oral Diseases &, Department of Oral and Maxillofacial Surgery, West China Hospital of Stomatology, Sichuan University, Chengdu, Sichuan China

**Keywords:** Tertiary lymphoid structures, Tumor microenvironment, Antitumor immunity, Immune checkpoint inhibitors, Prognostic biomarker, Immunotherapy

## Abstract

Tertiary lymphoid structures (TLS) are ectopic lymphoid aggregates formed under chronic inflammatory tumor microenvironments. Clinical cohorts across solid cancers demonstrate that mature TLS with intact germinal centers serve as independent favorable prognostic biomarkers and predict superior responses to immune checkpoint inhibitors. However, the differences in maturity, spatial localization and cellular composition of TLSs result in heterogeneity in their immunostimulation or immunosuppression, thereby exhibiting dual functions of promoting or anti-tumor. Furthermore, there are significant differences in the maturity assessment methods and definitions of TLS, which jointly hinder the clinical evaluation and targeted treatment of TLSs. In this review, we integrate current understanding of TLS development, molecular drivers, and stromal licensing, and dissect the key mechanisms by which TLS contribute to anti-tumor immunity within tumors. We consolidate standardized TLS detection methods encompassing pathology, multi-omics, and radiogenomics, and further explore the translational value of TLS maturation status, germinal center-like responses, and intratumoral versus peritumoral localization in prognosis and immune checkpoint blockade. We systematically elaborate therapeutic strategies for inducing functional TLS, dissect the intrinsic mechanisms by which TLS synergistically boost the efficacy of adoptive cell therapy and tumor vaccines, and outline major bottlenecks hindering clinical translation. Collectively, this work constructs a comprehensive multi-dimensional framework for TLS research and provides actionable insights to develop TLS-targeted immunotherapies for human cancers.

## Introduction

Tertiary lymphoid structures (TLSs) are ectopic lymphoid aggregates that arise in non-lymphoid tissues under conditions of persistent inflammation. TLSs share several structural and functional features with secondary lymphoid organs (SLOs), including B-cell follicles, T-cell-rich zones, fibroblastic reticular cell (FRC) networks, follicular dendritic cell (FDC) scaffolds, and high endothelial venules (HEVs) [1–3]. In tumors, TLSs are commonly located within the tumor parenchyma, at the invasive margin, or in the adjacent stromal compartment, where they function as local immune niches that support antigen presentation, lymphocyte recruitment, and the organized coordination of adaptive immune responses [1, 2, 4]. However, their establishment and maintenance are highly complex processes shaped by tumor-specific inflammatory signals and continuously remodeled by aberrant vasculature, fibrosis, metabolic stress, therapeutic exposure, and immunosuppressive factors within the tumor microenvironment (TME) [5–7].Across multiple solid tumors, the presence of TLSs has been associated with improved survival and a higher likelihood of response to immune checkpoint inhibitors. However, this association is not uniform across cancer types and is not independent of TLS maturity, anatomic location, or stromal context [5, 6, 8].

Although a large number of clinical cohorts have confirmed that TLS has a bidirectional regulatory effect, the existing reviews have not yet constructed a complete research system integrating the TLS development pathway, intratumoral immune mechanism, multi-dimensional detection methods and targeted treatment strategies [9–11]. Firstly, there is currently a lack of unified pathological assessment standards and standardized classification systems for describing the maturity and spatial distribution of TLS. Over the past decade, single-cell sequencing and spatial multi-omics technologies have greatly expanded our understanding of TLS, but the heterogeneity of TLS is still not fully understood [12–14]. In addition to, new strategies for regulating TLS-induced therapy have been experimentally confirmed [15, 16], yet lack in-depth elaboration of the intrinsic synergistic mechanisms of artificially constructed TLS combined with adoptive cell therapy and tumor vaccines.

This review systematically integrates the latest basic research on tumor TLS, multi-center clinical data, and preclinical treatment progress, establishing a comprehensive multi-dimensional review in the field of TLS. Firstly, we systematically analyzed the various inducing cells and stromal organizer cells involved in the initiation of TLS, as well as the signaling pathways; highlighting the heterogeneity of TLS within the tumor microenvironment. Secondly, analyze the bidirectional immune regulatory role of TLS from the perspectives of cells, space, and metabolism, and summarize the complete set of TLS detection technologies including pathological staining, multi-omics, artificial intelligence digital pathology, and radiomics. Finally, analyze the predictive value of TLS for patient prognosis and its molecular mechanism that mediates anti-tumor immunity, and systematically summarize various TLS-induced therapeutic approaches.

## Development and induction of TLSs in cancer

The development of TLSs in cancer is a multistep, highly coordinated spatiotemporal process. Their formation generally follows a peripheral cell-driven cascade in which persistent tumor antigens and chronic inflammation promote the emergence of alternative lymphoid tissue inducer-like (LTi-like) cells. These LTi-like cells then interact with LTo/stromal cells, release chemokines and adhesion molecules to establish lymphocyte recruitment circuits, and subsequently facilitate the formation of HEVs, which amplify cellular recruitment as TLSs progress through early, primary follicle-like, and secondary follicle-like mature stages.

### Key cellular initiators and organizers

TLS formation in tumors is often compared with lymph node organogenesis, which classically depends on interactions between embryonic lymphoid tissue inducer (LTi) cells and lymphoid tissue organizer (LTo) cells. In tumor tissues, however, classical embryonically derived LTi cells are absent, and TLS formation appears to rely instead on inflammation-driven alternative LTi-like programs rather than a strict recapitulation of embryonic development [1–3]. The mechanisms underlying this inflammation-driven lymphoid neogenesis vary across cancer types, such that different tumors may recruit distinct LTi-like cell populations. Nevertheless, these programs appear to converge on stromal activation, for example through expression of lymphotoxin LTα1β2, activation of lymphotoxin-β receptor (LTβR) on stromal and endothelial cells, and subsequent induction of HEV differentiation and TLS architectural assembly.

In lung cancer, NCR(+) ILC3s are preferentially localized at the periphery of tumor-associated TLSs, display clear lymphoid tissue-inducing properties, and can interact with tumor cells and cancer-associated fibroblasts; their abundance positively correlates with TLS density, suggesting that ILC3s represent one of the more typical LTi-like cell populations in non-small cell lung cancer [17, 18]. Similarly, in colorectal cancer, NKp44(+) ILC3s highly express TLS-associated genes including LTA, LTB, and TNF, and their numbers decline with tumor progression in parallel with TLS density, further indicating that ILC3s participate in a TLS initiation program linked to mucosal tissue contexts [19]. In contrast, TLS formation in pancreatic ductal adenocarcinoma appears to depend more heavily on an IL-33-driven ILC2 program: IL-33 activates lymphotoxin-expressing ILC2s and promotes their interaction with local LTβR(+) organizer cells, thereby initiating tertiary lymphoid neogenesis [20]. Taken together, these studies indicate that even within the innate lymphoid cell lineage, the TLS initiation modules mobilized by different cancers are not identical.

Th17 cells are another widely discussed population with alternative LTi-like activity, in part because they express the canonical LTi lineage transcription factor RORγt [21, 22]. By secreting cytokines such as IL-17 and IL-22, Th17 cells can establish a proinflammatory environment conducive to TLS neogenesis [23]. In addition, effector lymphocytes and activated B cells may also contribute to TLS initiation, amplification, and maintenance. Activated CD8-positive T cells and NK cells can provide lymphotoxin-family signals that promote fibroblast organization and support endothelial conversion toward an HEV-like state, particularly in treatment-induced immune activation settings [24]. Activated B cells can likewise express LTα1β2 and participate in the induction process [4]. In some contexts, early activated myeloid cells have also been observed to exert LTi-like effects [25, 26]. These populations are therefore better understood as context-dependent candidate or cooperative LTi-like programs rather than as a uniform initiating cell type.

Whether TLSs ultimately form also depends on the local presence of activatable organizer cells. LTo cells are stromal-derived cell populations that receive LTα1β2 signals from LTi cells or their substitutes through LTβR and subsequently differentiate into phenotypes with lymphoid chemotactic and supportive functions. Once activated, these cells produce chemokines such as CCL19, CCL21, and CXCL13, remodel the extracellular matrix, generate reticular migratory tracks, and establish a local environment that supports lymphocyte adhesion and compartmentalization. The best-defined local organizer populations currently include LTo-like CAF/FRC populations, LTβR(+) myeloid organizer cells, and LTβR-responsive endothelial cells; in inflammatory settings, pericytes and adipocytes have also been shown to possess LTo potential [27]. Fibroblasts from multiple origins can differentiate into cancer-associated fibroblasts (CAFs), acquire LTo-like features in response to tumor and inflammatory cues, form FRC networks, and thereby guide T- and B-cell migration [24, 28]. Endothelial cells can undergo phenotypic conversion in response to LTβR signaling and factors such as LIGHT, upregulate HEV markers, and acquire the ability to actively recruit lymphocytes [29, 30]. Studies in Sjogren syndrome models suggest that pericytes, similar to fibroblasts, may secrete CCL19 and CCL21 and directly recruit lymphocytes [31]. Adipocytes, meanwhile, can secrete adipokines and proinflammatory cytokines under specific inflammatory conditions to recruit and activate lymphocytes, thereby promoting TLS formation [32].

**Fig. 1:**
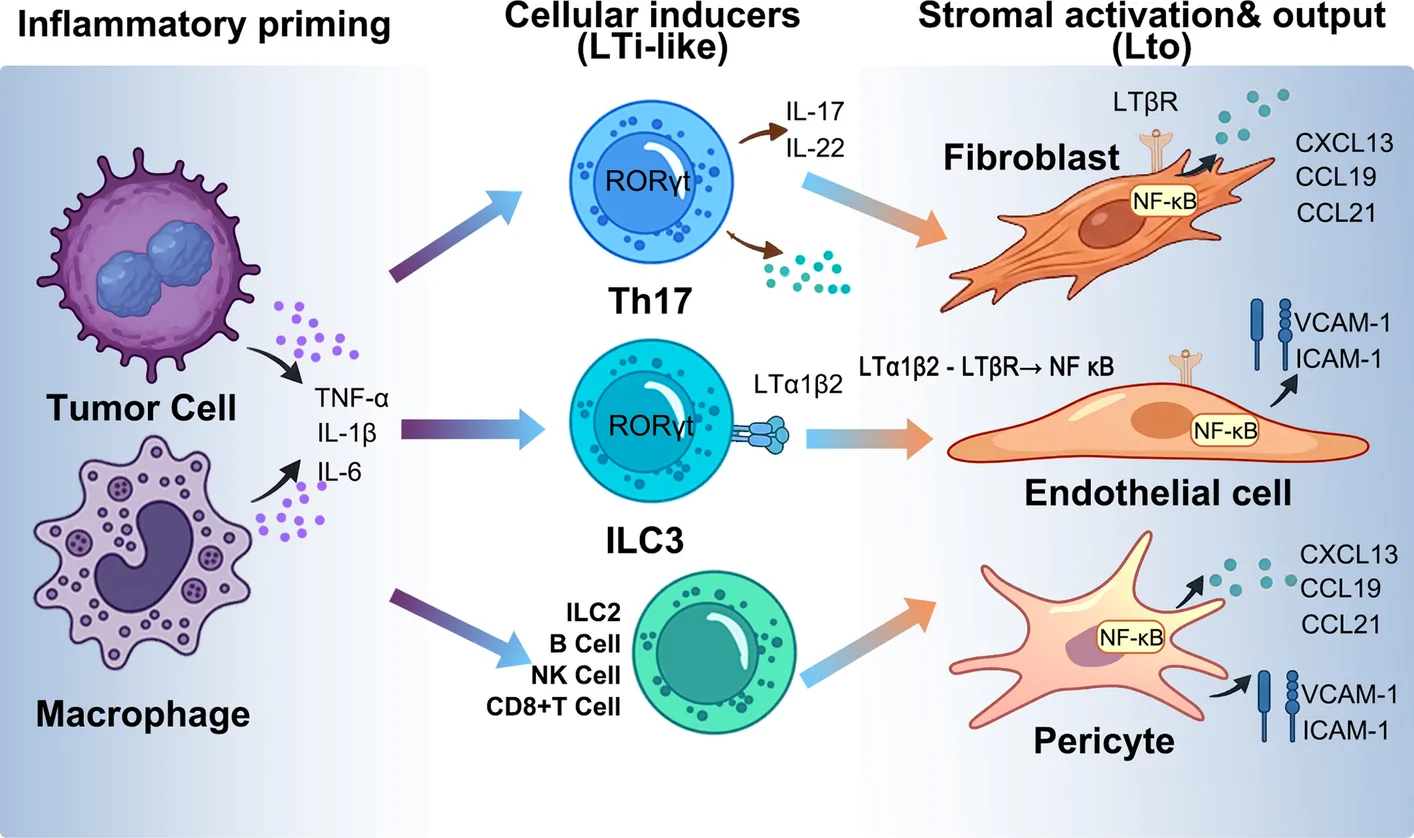
Canonical and alternative pathways of TLS initiation in cancer. Chronic inflammatory cues in the tumor microenvironment promote LTi-like immune programs that activate stromal organizer cells through the LTα1β2-LTβR-NF-κB axis. In response, stromal cells produce CXCL13, CCL19 and CCL21 and upregulate VCAM-1 and ICAM-1, thereby establishing a niche that supports immune-cell recruitment, retention and spatial organization for TLS neogenesis. An alternative IL-17-driven pathway can directly activate stromal cells independently of canonical lymphotoxin signaling, providing an additional route for early TLS induction

Taken together, tumor-associated TLSs are not linearly triggered by a fixed list of cells; rather, they are driven by inflammatory circuits composed of cancer type-specific LTi-like inducer cells acting in concert with local organizer cells. In other words, the truly generalizable feature of TLS initiation is not a single invariant cell type, but a mechanistic framework in which diverse inducer signals converge on local organizer programs and are transformed into an LTβR-centered lymphoid neogenesis circuit. Which cells assume inductive, amplifying, or maintenance roles, whether ILC2s, ILC3s, Th17 cells, CD8 T cells, NK cells, or B cells, depends on cancer type, organ niche, and treatment context. Successful TLS establishment therefore requires not only inducer cells but also stromal licensing, namely the capacity of local stromal compartments to respond to inflammatory signals and enter organizer programs. Some tumors may show evident inflammatory infiltration yet still fail to form mature TLSs when their stroma is highly fibrotic, their vasculature is aberrant, or immunosuppressive programs dominate [6, 9, 27] (Fig. 1).

### Molecular drivers and signaling pathways

Chronic inflammation in the TME provides the soil for TLS formation. Inflammatory mediators released by tumor or immune cells, including IL-1β, IL-6, and TNF-α, act as initiating signals that activate and recruit the alternative LTi-like cells described above [7, 33]. Although the identity of alternative LTi-like cells is heterogeneous, multiple studies consistently identify the canonical LTα1β2-LTβR axis as the core pathway driving TLS formation. Activated LTi-like cells express membrane-bound lymphotoxin α1β2 (LTα1β2), which binds LTβR on stromal or endothelial cells and activates both canonical and noncanonical NF-κB signaling pathways, thereby inducing downstream lymphoid chemokines such as CCL19, CCL21, and CXCL13, adhesion molecules including VCAM1, ICAM1, MAdCAM1, and PNAd, as well as vascular remodeling programs that initiate TLS assembly [34–36]. Pharmacologic LTβR agonism can enhance antitumor immunity and promote TLS-associated remodeling, whereas combined activation of STING and LTβR can further amplify B-cell responses within TLSs [35, 36]. In addition, IL-17, mainly produced by Th17 cells, can drive lymphocyte aggregation and organization even in the absence of lymphotoxin, thereby constituting an alternative route for TLS initiation [37–39].

Once stromal cells are activated, lymphocytes are recruited into TLSs on a large scale, and this spatial organization provides the structural basis for subsequent GC formation and antibody responses. Evidence from patient samples across cancer types and from experimentally induced models indicates that two chemokine systems are particularly important in this process [40–42]. The first is the CXCL13-CXCR5 axis, which is widely regarded as the core chemokine system for B-cell recruitment and follicle formation [40, 43]. CXCL13 is a potent chemoattractant for B cells and Tfh cells and, through binding to CXCR5, recruits these cells to the TLS center and promotes the formation of B-cell follicles and GCs [4]. The subsequent production of CXCL13 by FRC/FDC networks sustains the local gradient, supports B-cell differentiation and expansion, and ultimately enables the development of mature TLSs containing GCs [44, 45]. The second is the CCL19/CCL21-CCR7 axis. Through binding CCR7 on T cells and mature dendritic cells, CCL19 and CCL21 recruit T cells to the T-cell zone surrounding the B-cell follicle and guide DC migration into T-cell areas, thereby promoting T-cell activation and antigen presentation [9, 10, 19]. The CXCL13-CXCR5 and CCL19/CCL21-CCR7 axes not only establish B-cell follicular and T-cell zonal compartmentalization within TLSs, but their local expression strength, persistence, and stable anchoring within FRC/FDC-like stromal networks may also determine whether TLSs remain loose lymphocytic aggregates or progress into organized mature structures characterized by FDC networks, HEVs, and GC-like reactions [46, 47].

The transition of TLSs from early cellular aggregates to functionally mature structures is marked by the specialization and formation of HEVs. By expressing distinct adhesion molecules and chemokines, HEVs establish an efficient lymphocyte homing system [20]. Their significance lies not only in increasing cell entry, but also in providing the TME with a vascular interface that continuously replenishes new lymphocytes, thereby allowing otherwise transient immune aggregates to evolve into sustainable local immune niches [48–50]. Induction and maintenance of HEVs depend on an LTβR-centered differentiation program: LTα1β2 expressed by LTi cells activates LTβR on stromal and endothelial cells, driving endothelial conversion from a flattened phenotype to cuboidal high endothelial cells (HECs), whereas LIGHT (TNFSF14), expressed mainly by activated T cells, NK cells, and immature dendritic cells, acts as a complementary ligand [51]. LTβR on endothelial cells, stromal cells, and certain myeloid populations can activate both canonical and noncanonical NF-κB pathways, thereby providing the chemotactic and adhesive basis for local lymphoid organization [52]. LIGHT can also bind HVEM on T cells and deliver costimulatory signals, enabling multidimensional remodeling of the tumor immune microenvironment [34, 36, 53]. Functional HEVs express high levels of the sulfated sialomucin PNAd, which binds L-selectin (CD62L) on lymphocytes; other adhesion molecules, including MAdCAM1, ICAM1, and VCAM1, interact with lymphocyte integrins such as LFA1 and VLA4 to mediate firm adhesion and transendothelial migration [49, 54].

TLS formation is not driven solely by positive inductive cues, but is also constrained by multiple inhibitory tissue programs. Fibrosis and dense stroma can restrict lymphocyte entry, retention, and aggregation through the FAP + CAF-CXCL12-CXCR4 axis and through migratory barriers created by collagen and fibronectin accumulation, thereby weakening the foundation for nascent TLS formation [55, 56]. Hypoxia, through the HIF-1α-CD39/CD73-adenosine axis as well as through inhibition of DC maturation, CCR7-dependent migration, and IL-12 production, can further impair TLS organization and functional maturation [57, 58]. Persistent PMN-MDSCs and M2-like macrophages can suppress antigen-specific T-cell responses through mechanisms including ARG1 and are associated with immature TLS states [59, 60]. Taken together, the absence of TLSs or failure of TLS maturation is often not simply a matter of insufficient inductive signaling, but rather the combined result of structural, metabolic, and myeloid suppressive programs.

### Maturation stages and heterogeneity

At the most basic level, TLSs are ectopically formed lymphoid aggregates in chronically inflamed tissues that display a certain degree of organization. Traditionally, TLSs have been divided into early TLSs, primary follicle-like TLSs, and secondary follicle-like TLSs. This classification remains practical in pathology because it corresponds to a continuum of histologic changes ranging from simple lymphocyte aggregation to follicle formation and then to the emergence of GC-like reactions. At the same time, recent multiplex imaging and spatial transcriptomic studies indicate that TLS maturation is not a set of strictly discrete categories, but rather a dynamic continuum in which different TLSs gradually acquire FDC networks, HEV features, plasma cell output, and more complete spatial compartmentalization, whereas some immature TLSs may deviate from this trajectory under specific metabolic or inflammatory conditions [61, 62].

In practical pathologic assessment, H&E is usually used for initial screening to determine whether relatively circumscribed lymphocytic aggregates are present within the tumor or at the tumor margin, but morphology alone is often insufficient to define TLS maturity accurately. Additional immunomarkers can refine classification: CD20 and CD3 identify B- and T-cell compartmentalization; CD21 and CD23, with CD35 when necessary, indicate FDC networks [63]; BCL6, Ki67, and in some studies AID suggest GC-like reactions [9, 64]; and PNAd, commonly detected using the MECA-79 antibody, identifies HEVs. Importantly, the presence of HEVs supports a strong capacity for lymphocyte recruitment but does not in itself define a mature TLS. Compared with single-marker staining, multiplex immunofluorescence or multiplex immunohistochemistry allows simultaneous visualization of B/T compartmentalization, FDC networks, HEVs, and DC distribution on the same section, thereby reducing misclassification caused by serial sections. Spatial transcriptomics can further identify chemokine programs, plasma cell output, and stromal organizer programs.

**Fig. 2:**
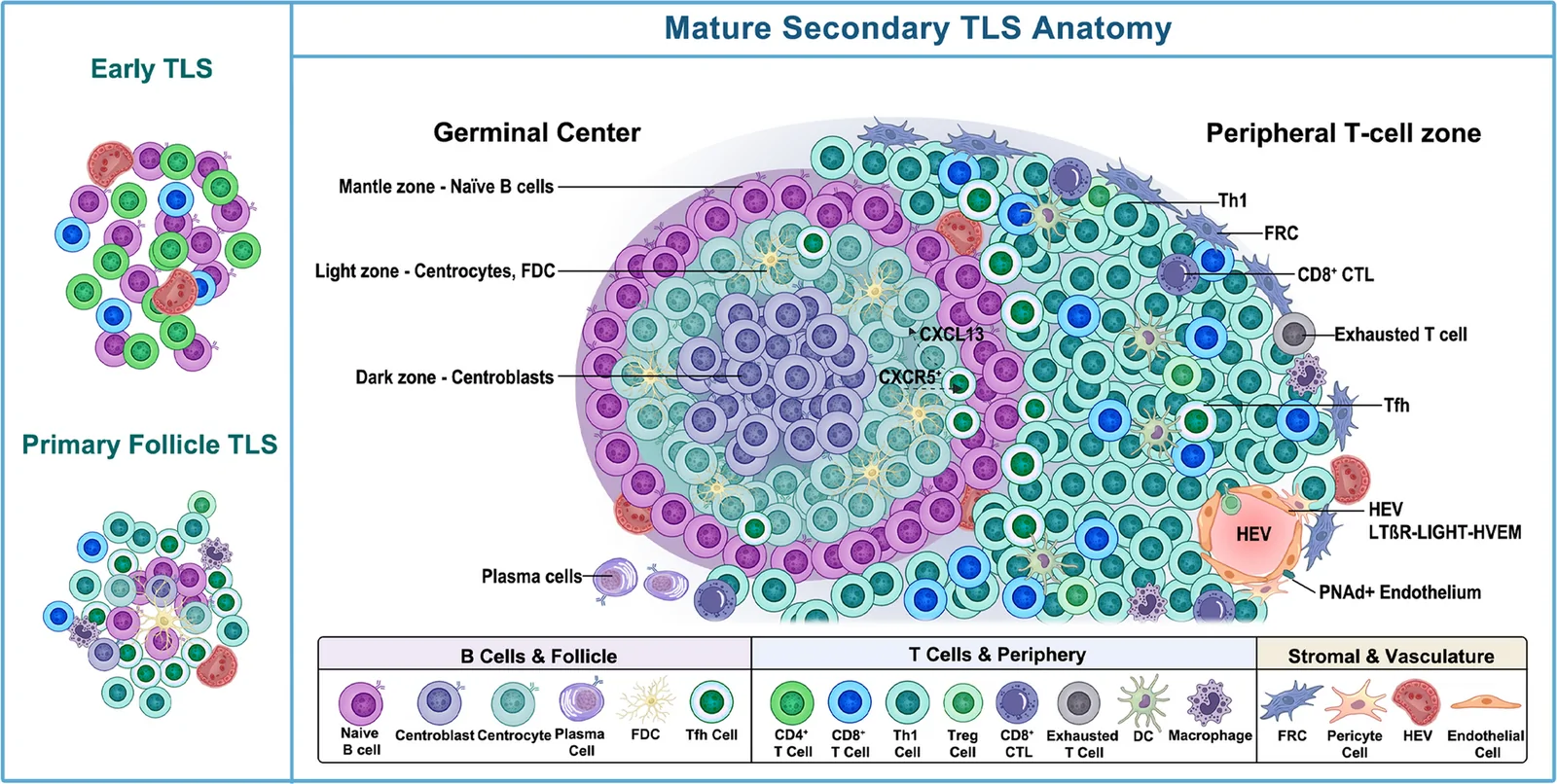
Progressive maturation of TLS in cancer. The schematic illustrates the stepwise maturation of tertiary lymphoid structures (TLS) in cancer, from early TLS and primary follicle TLS to fully mature, germinal centre-containing TLS. Early TLS are characterized by loose lymphoid aggregates with limited compartmentalization, whereas primary follicle TLS display a more defined follicular organization. Mature TLS exhibit clear segregation of B-cell and T-cell zones, follicular polarization, specialized stromal networks and HEV-supported lymphocyte recruitment, together enabling local immune-cell organization and activation

Within this framework, early TLSs usually appear as relatively loose lymphocytic aggregates with absent or only partial B/T compartmentalization and without a clear FDC network. Primary follicle-like TLSs exhibit more evident B-cell clusters and adjacent T-cell zones, together with CD21 + FDC networks, but still lack clear GC reactions. Secondary follicle-like or mature TLSs are defined by the appearance of GC-like reactions and usually display clear B/T spatial segregation, CD21 +/CD23 + FDC networks, and BCL6 +, often Ki67 +, GC-like B-cell populations; in some studies AID has also been detected to support ongoing somatic hypermutation and class-switch recombination programs. Mature TLS T-cell zones often contain DC-LAMP + or CD83 + mature dendritic cells, Tfh cells, and lymphocyte influx routes associated with PNAd + HEVs [62, 65]. Importantly, mature TLSs in tumors do not necessarily recapitulate the classic dark zone/light zone polarity of secondary lymphoid organs. For example, in melanoma, many secondary follicle-like TLSs possess CD21 +/CD23 + FDC networks and GC-like structures but still lack classical GC polarity. It is therefore more accurate to describe them as mature TLSs with GC-like reactions rather than to require strict equivalence to lymph node follicles. Whether TLSs can progress to this mature state is itself highly dependent on cancer type and lesion location. Even in tumors capable of forming TLSs, TLS maturity may vary substantially across patients, lesions, and spatial positions inside and outside the tumor. Under strongly immunosuppressive conditions, TLSs often remain in early or primary follicle-like stages, characterized by absent GCs, a scarcity of mature dendritic cells, T-cell exhaustion, and enrichment of suppressive myeloid cells [9, 33, 66, 67] (Fig. 2).

In addition to temporal heterogeneity, TLS maturation is also spatially heterogeneous. TLSs are not isolated structures in a two-dimensional histologic plane. Recent studies based on whole-slide multiplex immunofluorescence and prior three-dimensional reconstruction suggest that TLSs more closely resemble multilobular, interconnected three-dimensional networks. Accordingly, the apparent TLS domain seen on a single section can vary substantially in size, morphology, and whether a GC-like region is captured, depending on the plane of sectioning, which in turn affects assessment of TLS number and maturity [68]. Moreover, TLSs located within the tumor, at the invasive margin, or in peritumoral regions are not biologically equivalent, because they differ in opportunities for contact with tumor antigens, the stromal barriers they encounter, vascular state, and the nearby suppressive myeloid cell populations [6, 9].

Intratumoral TLSs are closer to the source of tumor antigens and are therefore more often associated with local immune activation, favorable prognosis, and improved immunotherapy responses. In head and neck squamous cell carcinoma, intratumoral TLSs are associated with better survival and immunotherapy response [69], whereas in cholangiocarcinoma, a high density of intratumoral TLSs correlates with longer overall survival [70]. TLSs at the invasive margin function more as interface sites between the tumor and the host immune system, and their value depends on whether they can form effective spatial coupling with the tumor parenchyma. Spatial omics in recurrent or metastatic head and neck squamous cell carcinoma has shown that the greater the distance between TLSs and tumor cells, the worse the outcome with immune checkpoint inhibition [71]. Peritumoral or para-cancerous TLSs are the most context-dependent. In some cancer types they may reflect active peripheral immune priming, whereas in other settings characterized by strong stromal barriers and enriched myeloid suppression they may instead represent a spatially constrained immune-excluded response, in which immune cells accumulate around the tumor but fail to penetrate the tumor parenchyma effectively [72, 73]. TLSs embedded in immune-supportive stroma are therefore functionally distinct from those situated in fibrotic, TGF-β-dominant, or vascularly abnormal environments.

## Cellular and spatial composition of TLSs

### Structural organization

Mature TLSs are highly compartmentalized microanatomic units. Their typical organization includes a central B-cell-rich follicular area, surrounding T-cell-dominant zones, and interspersed HEVs, dendritic cells, and stromal scaffolds that mediate continuous cellular trafficking and information exchange [1, 4]. In TLSs containing GCs, the B-cell compartment is further subdivided into dark and light zones that correspond to successive steps of B-cell proliferation, somatic hypermutation, selection, and differentiation, and these zones are enclosed by a mantle zone [74, 75]. The dark zone is mainly composed of rapidly proliferating centroblasts, whereas the light zone is enriched for centrocytes and FDCs. The mantle zone consists of resting naive B cells and serves as a reserve pool that can replenish GC responses [76, 77].

The T-cell zone is supported by a three-dimensional network formed by FRCs, which provides the structural basis for T-cell migration and survival. This region contains multiple T-cell subsets, including CD8 + and CD4 + T cells [78, 79]. HEVs are a key vascular component of TLSs and are mainly distributed within the T-cell zone, particularly at the T-B border and in peripheral TLS regions [49].

### Core cellular components

Single-cell studies have identified multiple B-cell states in tumor-associated TLSs across cancer types, including naive B cells, GC B cells, memory-like B cells, plasmablasts, and plasma cells. However, the relative abundance and distribution of these states vary substantially with cancer type and TLS maturity [62, 80]. Naive B cells enter TLSs from the circulation through HEVs. After encountering antigen and receiving helper signals at the T-B border, they may initiate GC reactions or follow extrafollicular differentiation pathways. Activated B cells that enter the GC program differentiate into centroblasts, upregulate CXCR4, localize to the dark zone, and undergo rapid proliferation and somatic hypermutation. Some centroblasts then exit the cell cycle, migrate to the light zone, and differentiate into centrocytes with reduced CXCR4 expression; there they undergo antigen-dependent selection on FDCs and, with Tfh help, complete affinity maturation and class switching before differentiating into plasma cells or memory B cells.

TLSs can be regarded as local factories of humoral immunity rather than simple reservoirs of lymphocytes, and B-cell programs may diverge into protective or non-protective trajectories depending on the local immune and stromal environment. In several cancer types, TLS-rich tumors are accompanied by a more IgG-skewed humoral immune program, which is associated with better prognosis, stronger antigen-presenting capacity, and improved responses to immune checkpoint inhibition [80, 81]. In other settings, however, IgA-skewed plasma cell programs, atypical memory B-cell states, or immunoregulatory Breg phenotypes can also be observed; these programs are more likely to reflect chronic stimulation, tissue remodeling, or immunosuppression and do not necessarily indicate effective antitumor immunity [82, 83].

T-cell populations in TLSs are likewise highly diverse and functionally central. CD4 + T cells located within or around TLSs may include Th1 cells, Tfh cells, conventional helper T cells, regulatory T cells, and, in some cancer types, progenitor-exhausted or stem-like populations. Together, these subsets determine the extent of B-cell help, cytokine production, and the quality of local adaptive immunity [66, 84, 85]. CD8 + T cells, by contrast, may exhibit recently recruited naive or central-memory-like states, activated cytotoxic states, or TCF1-positive stem-like states with proliferative potential [47, 85]. The relative abundance and spatial localization of these different states likely determine whether TLSs preferentially support acute cytotoxic responses, durable memory responses, or inefficient chronic stimulation.

**Fig. 3:**
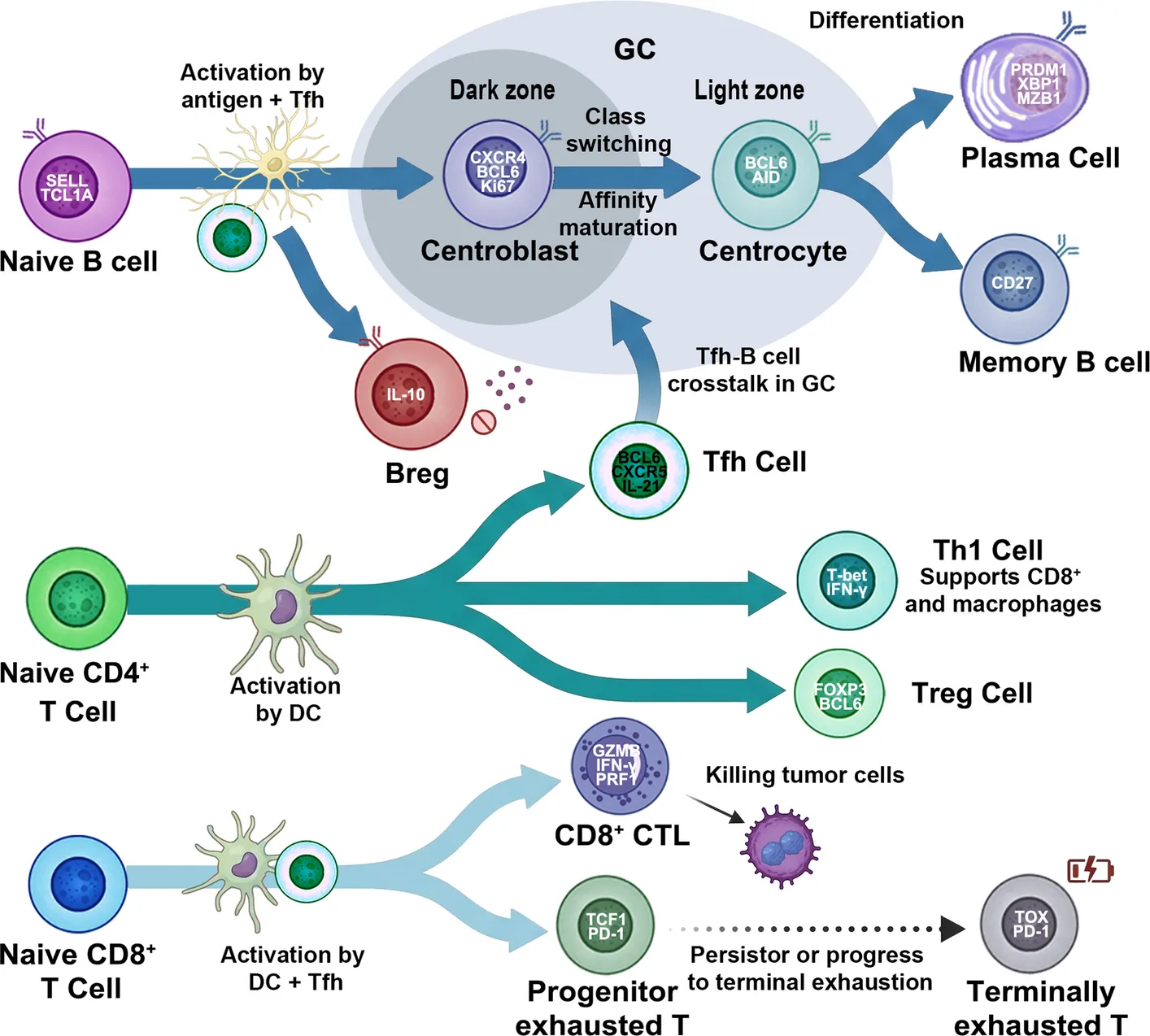
Developmental trajectories of B and T cell lineages within tertiary lymphoid structures. Schematic of the major differentiation states of B cells and T cells in TLS. Naïve B cells activated by antigen and Tfh cells enter the germinal-centre-like reaction and differentiate into centroblasts, centrocytes, plasma cells, memory B cells or IL-10-producing Breg cells. Naïve CD4 + T cells differentiate into Tfh, Th1 and, optionally, Tfr cells, whereas naïve CD8 + T cells give rise to effector cytotoxic T cells or progenitor exhausted T cells that may progress to terminal exhaustion

After activation by mature dendritic cells, naive CD4 + T cells upregulate CXCR5 and downregulate CCR7, gain the capacity to migrate toward the B-cell compartment, express BCL6, and differentiate into Tfh cells, thereby occupying a central position that links humoral and cellular immunity. In addition to supporting GC reactions, IL-21 secreted by Tfh cells can act on CD8 + T cells to upregulate granzyme B (GZMB) and IFN-γ, thereby promoting effector differentiation and cytotoxic function [65]. Th1 cells differentiate in the presence of IL-12 and IFN-γ, express T-bet, secrete IFN-γ, and support activation of CD8 + T cells and macrophages. In recent years, TCF1-positive stem-like or progenitor-exhausted T cells have attracted particular attention because they can maintain self-renewal under chronic antigen stimulation and immune checkpoint blockade while generating more differentiated effector progeny [84, 86]. In head and neck squamous cell carcinoma, mature TLSs are associated with enhanced intratumoral T- and B-cell responses, and part of this effect appears to be mediated by progenitor-exhausted CD4-positive T cells, suggesting that TLSs support not only terminal effector states but also stem-like and intermediate differentiation states [66]. Thus, the functional significance of TLSs cannot be captured simply by counting T cells; what matters is whether the TME supports repeated antigen presentation, maintenance of TCF1-positive states, and helper-effector cooperation within an organized niche (Fig. 3).

Myeloid and stromal antigen-presenting networks are required for TLS function. Mature dendritic cells, particularly the DC-LAMP-positive or LAMP3-associated migratory dendritic cell states frequently observed in human tumors, are usually enriched in T-cell zones and at the T-B border, where they mediate antigen presentation, attract T cells, and sustain local activation programs [5, 6, 85]. The presence of mature dendritic cells is often associated with greater TLS maturity and stronger local immune activity.

FDCs are the defining component of the follicular compartment. They are not migratory antigen-presenting cells but specialized stromal cells that capture and retain immune complexes within B-cell follicles for prolonged periods. Through persistent display of native conformational antigens, they support affinity-dependent B-cell selection and the maintenance of GC reactions [44]. Consequently, histologic detection of an FDC network is often one of the most important indicators that a TLS has progressed from an early aggregate to a more mature functional structure [1, 5, 67]. In many studies, the presence of FDC-positive follicles has been used to distinguish prognostically meaningful mature TLSs from lymphoid aggregates that are only morphologically similar.

Discussion of macrophages within and around TLSs should move beyond the traditional M1/M2 dichotomy. Depending on their state, tumor-associated macrophages may participate in apoptotic cell clearance, tissue remodeling, regulation of vascular permeability, T-cell suppression, and antibody-dependent effector functions [77, 78, 87]. Some macrophage programs may cooperate with antibodies produced by plasma cells to enhance tumor cell clearance, whereas others may promote matrix remodeling, angiogenesis, or local immunosuppression and thereby weaken TLS stability [6]. For example, in hepatocellular carcinoma, MMP9 + tumor-associated macrophages are enriched at the invasive front and are associated with poor prognosis [86].

### Supportive stromal cells

The formation and function of TLSs depend heavily on stromal cells that provide architecture, chemokines, and survival signals. Fibroblasts, FRC-like cells, endothelial cells, and mesenchymal populations jointly establish chemokine gradients, migratory tracks, matrix structures, and lymphocyte entry routes, thereby allowing TLSs to persist [17, 19].

Cancer-associated fibroblasts are a complex and dynamic cell population within the TME and can exert tumor-promoting, immunosuppressive, or immunostimulatory functions [88]. Traditionally, CAFs, such as TGF-β-secreting subsets, have been thought to restrict T-cell infiltration, thereby potentially offsetting the antitumor effects of TLSs and influencing responses to PD-1 blockade [74]. For example, in neurally invasive pancreatic ductal adenocarcinoma, myofibroblastic CAFs (myCAFs) encircle tumors to form a fibrotic barrier, accompanied by high expression of T-cell exhaustion markers such as PD1 and TIM3 and by the absence of TLSs [89]. However, recent studies have also identified CAF subsets that support TLS formation. In lung cancer, CCL19 + CAFs differentiate into two subpopulations, perivascular reticular cells and T-cell zone reticular cells, which respectively recruit and guide T cells into tumors and support T-cell proliferation and effector function [17]. In gastric cancer, a functional immunosupportive CAF subset around TLSs, termed apCAF, promotes T-cell activation and antitumor immunity through MHC class II-mediated antigen presentation [90]. In colorectal cancer, inflammatory CAFs with a CCL19 + phenotype attract lymphocytes through the CCL19-CCR7 axis and promote their organization into structured TLSs [19].

HEVs are a hallmark structure of TLSs. As specialized conduits for lymphocyte entry into local tissues, HEVs continuously replenish immune cells and, in treatment settings, support the formation of niches enriched in stem-like T cells [48–50]. By expressing adhesion molecules such as peripheral node addressin (PNAd), HEVs mediate the rolling, adhesion, and migration of lymphocytes, particularly naive lymphocytes, into TLSs. Changes in HEV density or morphology directly influence the degree of local immune activation [9, 31].

Mesenchymal stromal cells (MSCs) and tumor-associated MSC programs are currently best understood as regulatory factors at the margins of the TLS organizer landscape. They may provide nutritional support, extracellular matrix scaffolding, or precursor states for TLS-associated stroma. Normal MSCs (nMSCs) are more enriched for molecules such as CXCL12, IL7, and PDGFRB that are associated with B-cell support and follicular-like stromal phenotypes, suggesting that they may favor B-cell activation and the establishment of a TLS-supportive microenvironment [91]. As tumors progress, MSCs can be cancer-educated into tumor-associated MSCs (CAMSCs), which highly express WT1, CD200, and stromal genes linked to fibrosis and poor prognosis and may therefore attenuate the functional benefits conferred by TLSs [9, 92].

## Detection and analytical approaches for TLS

The detection of tertiary lymphoid structures (TLS) in tumors utilizes histopathology as the gold standard, combined with molecular, spatial omics, and artificial intelligence technologies, to form a comprehensive detection system ranging from qualitative identification to quantitative assessment, and from morphology to function.

### Histological methods: hematoxylin and eosin staining, immunohistochemistry

Hematoxylin and Eosin (H&E) staining remains the gold standard for the detection of TLS infiltration. It highlights dense aggregates of lymphocytes, allowing direct observation of the structural morphology and maturation status of TLS. However, it cannot distinguish the maturity and cell subtype of TLS, leading to potential misdiagnosis and is only suitable for initial screening. Therefore, Immunohistochemistry (IHC) has become an important method for detecting TLS. Currently, it is generally accepted that aggregates of CD20⁺ B cells with a diameter greater than 150 μm serve as the minimal structural unit of TLS [10, 75]. To differentiate TLS from non-structural lymphocyte infiltration, multiple studies co-stain CD20 with T-cell markers (such as CD3, CD4, or CD8) to delineate the spatial arrangement of B-cell and T-cell areas [93–95]. In addition, follicular dendritic cells (FDCs, markers CD21, CD23, CD35) are used to identify primary follicular/immature TLS, while high endothelial venules (HEVs, markers PNAd/MECA-79) can indicate mature TLS [96]. Importantly, the presence of at least one DC-LAMP⁺ mature dendritic cell (DC) structure in TLS suggests its functional activity, and Ki-67 reflects its germinal center activity [97] (Table 1).

**Table 1:** Core marker combination of TLS

Biomarker	Labeled cells	Purpose
CD3	Total T cells	Define Area T
CD20	B cells	Define Area B
CD21/CD23	Follicular dendritic cells (FDC)	Determine primary follicles/immature TLS
PNAd(MECA-79)	High endothelial microveins (HEV)	Determine primary follicles/immature TLS
DC-LAMP	Mature dendritic cells	Prompt function activity
Ki-67	proliferative cells	Determine the activity of the germinal center

In addition to basic morphological analysis, Multiplex Immunofluorescence (mIF) has been established as the gold standard in scientific research for conducting a comprehensive assessment of the spatial architecture and cellular interactions within the Tumor-Lymphoid-Stromal (TLS) microenvironment. In mechanistic investigations of diverse tumors, including Non-Small Cell Lung Cancer (NSCLC), cervical cancer, Pancreatic Ductal Adenocarcinoma (PDAC), Hepatocellular Carcinoma (HCC), and ovarian cancer, mIF enables simultaneous visualization of the spatial distribution of T/B/DC/FDC/HEV cells, precisely reconstructing the three-dimensional structure of TLS with unparalleled spatial resolution [20, 75, 98, 99]. Additionally, Imaging Mass Cytometry (IMC) can be utilized for refined subtyping of the TLS microenvironment, identification of suppressive TLS (characterized by Treg/Breg enrichment), and delineation of stem cell immune hubs [75, 100].

### Transcriptomic signatures

Transcriptomics is the most mature and widely applied strategy for detecting TLS molecules at the molecular level in tumor tissues. By identifying the specific expression profile of TLS, it enables quantitative assessment of its presence, abundance, and maturation status, effectively compensating for the subjectivity and limitations of traditional morphological detection. It provides high-dimensional analysis of the composition, maturity, and heterogeneity of TLS in different cancer types. Currently, multiple cross-cancer type general and tumor-specific TLS transcriptomic features have been established. Among them, the 12-chemokine gene signature (CCL2, CCL3, CCL4, CCL5, CCL8, CCL18, CCL19, CCL21, CXCL9, CXCL10, CXCL11, CXCL13) is considered as the core molecular marker for identifying TLS, which can stably recognize the formation of ectopic lymphoid structures within tumors [101, 102]. CXCL13, as a key chemokine regulating B cell recruitment and TLS maturation, is the most specific single marker, and its expression level is closely related to TLS density, germinal center formation, and immunotherapy response [103, 104]. Based on this, researchers further developed a mature 29-gene set specific to tumor-infiltrating lymphocytes (TLS), a 4-gene classifier for cholangiocarcinoma (PAX5, TCL1A, TNFRSF13C, CD79A), and gene characteristics related to the germinal center of pancreatic cancer (AICDA, IL21R, CD79A), etc. These can significantly enhance the objectivity and accessibility of TLS assessment through techniques such as qPCR, NanoString, and whole transcriptome sequencing [12, 104, 105]. The TLS score based on transcriptome has been validated in various tumors such as colorectal cancer, hepatocellular carcinoma, and urothelial carcinoma, and can quantify TLS activity and independently predict the efficacy of immune checkpoint inhibitors, providing standardized molecular indicators for clinical stratification [106, 107].

The rapid development of spatial multi-omics technology has advanced TLS molecular detection from the whole tissue level to the in situ spatial analysis level, enabling the simultaneous characterization of structural features, molecular phenotypes, and functional states, and becoming the core technology for precisely analyzing TLS heterogeneity. Spatial transcriptomics (Visium, Stereo seq) and spatial proteomics (GeoMX DSP) can accurately capture the molecular expression profiles of TLS in different regions within the tumor, at the invasive edge, and adjacent to the tumor, distinguishing between functionally mature TLS, inhibitory TLS, and regressive TLS [9]. Mature secondary follicular-like TLS (SFL TLS) highly expresses genes related to germinal center reactions such as BCL6, AICDA, and CXCR5, while immunosuppressive TLS is enriched in regulatory molecules such as FOXP3, IL-10, and TGFB. The differences in spatial distribution and molecular characteristics directly determine their regulatory effects on tumor immunity [108, 109]. In addition, non-invasive molecular detection based on peripheral blood is emerging as a new direction. By detecting the expression of TLS-related molecules such as circulating CXCL13 and CCL19, the state of TLS within the tumor and the response to immunotherapy can be dynamically monitored, providing possibilities for non-invasive real-time assessment [110]. In summary, molecular detection has formed a multi-level technology system ranging from batch transcriptome quantification to spatial in situ analysis, and from tissue detection to non-invasive liquid biopsy, providing key technical support for the basic research and clinical translation of TLS.

### The application of digital pathology and deep learning in TLS automated detection

Digital pathology slides (WSI) combined with artificial intelligence deep learning technology have effectively addressed the bottlenecks of traditional pathological assessment, such as strong subjectivity, poor reproducibility, and difficulty in large-scale retrospective analysis. Fully automated TLS detection models based on H&E stained slides have been validated in various solid tumors such as colorectal cancer, soft tissue sarcoma, and non-small cell lung cancer. The mainstream algorithms utilize architectures such as ResNet, U-Net, and Mask RCNN. These methods enable objective and high-throughput analysis, but still require validation by pathologists and support from standardized experimental procedures [111]. In multi-center cohorts, AI models have demonstrated higher accuracy, sensitivity, and specificity in identifying TLS compared to manual interpretation by pathologists [112]. Further integration with deep learning models for immunohistochemistry or multiplex immunofluorescence images allows for simultaneous analysis of CD4 +/CD8 + T cells, B cells, FDC networks, and HEV distribution within TLS, enabling joint quantification of structure and cellular components [75, 105]. Currently, AI-based TLS automatic scoring systems have been proven to independently predict patient prognosis and the efficacy of immune checkpoint inhibitors [70, 113].

### Radiomics non-invasively predicts TLS characteristics

Radiomics extracts high-dimensional features from medical images to non-invasively infer TLS distribution. In hepatocellular carcinoma (HCC), a transfer learning-based MRI model integrating ResNet50-derived deep features and conventional texture descriptors achieves stable predictive performance (AUC = 0.85) [110]; another MRI study incorporating peritumoral features predicts TLS density with high accuracy (AUC = 0.91) [107]. In lung adenocarcinoma (LUAD), CT radiomics combined with deep learning-based segmentation identifies 18 TLS-associated imaging features (e.g., margin sharpness, heterogeneity), and a support vector machine (SVM) classifier shows robust performance across multicenter cohorts (internal validation AUC = 0.804, external validation AUC = 0.747) [114]. Radiomics signals are highly correlated with TLS abundance. The core mechanism lies in the fact that dense areas of TLS often accompany interstitial inflammation, vascular remodeling, and immune cell infiltration, which can form quantifiable imaging phenotypes on CT/MRI [115]. Although radiomics cannot directly distinguish the maturity of TLS, it can complement digital pathology and molecular testing to construct an integrated TLS evaluation system of "imaging-pathology-molecular", which holds significant clinical translational value [116].

In summary, an integrated technical system has been established for the detection of tertiary lymphoid structures, with histopathology as the gold standard, molecular detection as a functional supplement, spatial multi-omics for precise analysis, digital pathology and deep learning as standardized tools, and radiomics as a noninvasive extension. These technologies collectively propel TLS from basic immune mechanism research towards clinical practice in tumor prognostic stratification, immunotherapy efficacy prediction, and individualized treatment plan formulation (Table 2).

**Table 2:** Comparison of TLS detection methods

Method	Applicable scenarios	Core advantage	Limitations
H&E	Preliminary screening, retrospective large sample	Fast and low-cost	no classification and prone to misjudgment
IHC	Clinical routine and maturity determination	standardization	The information of individual labels is limited
mIF	Precise spatial structure	Multi-label co-positioning and visualization	higher cost
transcriptome	Batch screening and puncture of samples	Objective and high-throughput	No spatial information
AI + Digital Pathology	Standardization of large queues	Objective and repeatable	Training data required
Radiomics	Non-invasive preoperative assessment	non-invasive	indirect predictor

## Prognostic value of tertiary lymphoid structures

TLS, as functionally competent immune hubs that form ectopically within the tumor microenvironment, collectively determine the prognostic value across different cancer types and the predictive efficacy of immunotherapy through their abundance, maturity, spatial location, and cellular composition. Recent evidence indicates that mature TLS are significantly associated with good survival in most solid tumors. However, influenced by tumor type, molecular subtype, disease stage, and microenvironment remodeling, they can exhibit neutral or even adverse prognoses. Simultaneously, TLS, with their stable predictive ability independent of PD-L1 and TMB, have become core biomarkers for evaluating the efficacy of immune checkpoint inhibitors (ICIs). Their clinical translational prospects are clear, yet they still require the support of a standardized evaluation system.

### Correlation with improved patient survival: evidence from solid tumors

In cross-cancer clinical studies, mature, high-density, and intratumoral-localized TLS are stable and independent favorable prognostic markers, significantly correlated with longer OS, DFS, and PFS, constituting the common protective immune feature of solid tumors [63, 99, 117, 118]. In non-small cell lung cancer, the abundance of TLS and the density of internal DC-LAMP + mature dendritic cells (DCs) and CD20 + B cells are independent prognostic factors. Mature TLS can reduce the risk of death by approximately 70% and can "authorize" CD8 + T cells to exert a protective effect [118, 119]. In colorectal cancer, mature TLS containing germinal centers and FDC networks are directly associated with a lower risk of recurrence, and their predictive power is significantly superior to that of simple TLS counting [120, 121]. In hepatocellular carcinoma, intratumoral mature TLSs are closely associated with a reduced risk of early recurrence and can independently indicate a favorable prognosis [75]. The spatial distribution and density of TLS can also predict the clinical outcomes of patients with intrahepatic bile duct carcinoma. In addition, in solid tumors such as melanoma, soft tissue sarcoma, renal clear cell carcinoma, esophageal squamous cell carcinoma, bile duct cancer, endometrial cancer, and ovarian cancer, high abundance and structurally intact TLS have been proven to be associated with better long-term prognosis [62, 96, 120, 122–124]. The core mechanism is that SFL-TLS can form clear T/B zones, functional hair growth centers and HEV, mature TLS can efficiently initiate in situ humoral and cellular immunity, promote CD8 + T cell activation, plasma cell secretion of anti-tumor antibodies, and form sustained immune surveillance.

### Context-dependent associations

The prognostic value of TLS is highly context-dependent. The presence of immature structures, peritumoral localization, enrichment of immunosuppressive cells, and abnormal tumor-driven pathways can render it ineffective and even indicate a poor prognosis [12, 125]. In renal clear cell carcinoma, TLS is positively correlated with the activation of the PI3K–mTOR pathway, and is enriched in Treg and Breg, leading to poor prognosis for patients [126]; in hepatocellular carcinoma, peritumoral immature TLS is associated with neutrophil infiltration and chronic inflammation, suggesting immune dysregulation and worse prognosis [101, 127]; in breast cancer, a large number of peritumoral immature TLS is associated with higher tumor grade, increased risk of invasion and metastasis, and significantly shortened DFS and OS [128, 129]. In advanced lung cancer and head and neck squamous cell carcinoma, highly infiltrative Treg and Breg cells within the tumor-associated lymphocyte (TLS) directly impair the function of CD8 + T cells, rendering TLS devoid of protective value [130, 131]. Similarly, in EGFR-mutant lung cancer, the synergistic effect of Tfh-B cells within TLS is compromised, preventing the formation of effective anti-tumor immunity and completely eliminating prognostic value [132]. Deviating TLS in liver cancer is regulated by TDO2-kyurine metabolism, which hinders B cell differentiation and leads to immune incapability and a poor prognosis [12]. In head and neck squamous cell carcinoma (HNSCC), the density of TLS is consistent with the trend of B-cell infiltration, but it is not an independent predictor, while the levels of tumor and peripheral blood B-cells are more predictive [133]. Tumors lacking such a niche exhibit poor CD8 T-cell infiltration. In high-grade serous ovarian cancer (HGSOC), TLS is highly prevalent in omental metastatic foci but underdeveloped in primary ovarian foci, suggesting site-dependent function [134]. Therefore, the prognostic significance of TLS is not determined by its presence or absence, but rather by its maturation status, spatial localization, immune cell balance, and tumor genetic background.

### TLS as a predictive biomarker for immunotherapy response

TLS, with their unique advantages in maturity, spatial positioning, and cellular composition, have become a highly specific immune checkpoint inhibitor (ICI) efficacy predictive marker, independent of PD-L1, tumor mutational burden (TMB), and CD8⁺ tumor-infiltrating lymphocytes [96, 135]. This conclusion has been validated across multiple cohorts in various solid tumors, including melanoma, non-small cell lung cancer, soft tissue sarcoma, urothelial carcinoma, hepatocellular carcinoma, and bile duct cancer [10, 81, 93, 122, 136–140]. Mature TLS (SFL-TLS), equipped with a complete germinal center, follicular dendritic cell (FDC) network, and HEV structure, can efficiently mediate tumor antigen presentation, B-cell affinity maturation, in situ activation, and clonal expansion of effector T cells, serving as the core immune hub driving the ICI response. However, patients with only early/primary TLS (E-TLS/PFL-TLS), inhibitory TLS, or deviated TLS cannot significantly benefit from immunotherapy. Multiple large-scale studies have confirmed that patients with high-density enrichment of mature tumor-infiltrating lymphocytes (TILs) exhibit significantly improved objective response rate (ORR), progression-free survival (PFS), and overall survival (OS) after treatment with PD-1/PD-L1 inhibitors [96]. Furthermore, this predictive efficacy is not affected by the level of PD-L1 expression, effectively compensating for the stratification defects of traditional biomarkers. In the context of neoadjuvant immunotherapy, an increase in the number of TLS and their enhanced maturity after treatment are significantly positively correlated with the rate of pathological complete response, making them key indicators for early efficacy evaluation [141]. From a molecular perspective, the transcriptomic characteristics of TLS, centered around CXCL13, CCL19/CCL21, and 12—chemokines, enable noninvasive quantitative assessment of the functional status of TLS, further enhancing prediction accuracy [102, 104]. Simultaneously, the abundance and spatial interaction of B cells, Tfh cells, and plasma cells within TLS constitute the key cytological basis determining predictive value. It is worth noting that the predictive value of tumor-infiltrating lymphocytes (TILs) is context-dependent. Peritumoral immature TILs, inhibitory TILs enriched in regulatory T cells (Tregs) and B cells (Bregs), and deviant TILs associated with metabolic disorders not only lack predictive power but may also indicate resistance to immunotherapy. In summary, a standardized evaluation system for TILs that integrates maturity classification, spatial localization, cellular composition, and molecular characteristics has become an important direction for breaking through the bottleneck of precise stratification in immunotherapy, providing a novel and reliable biological basis for individualized immunotherapy decision-making.

## Mechanistic drivers of anti-tumor immunity within TLS

### The local adaptive immune synapse

**Fig. 4:**
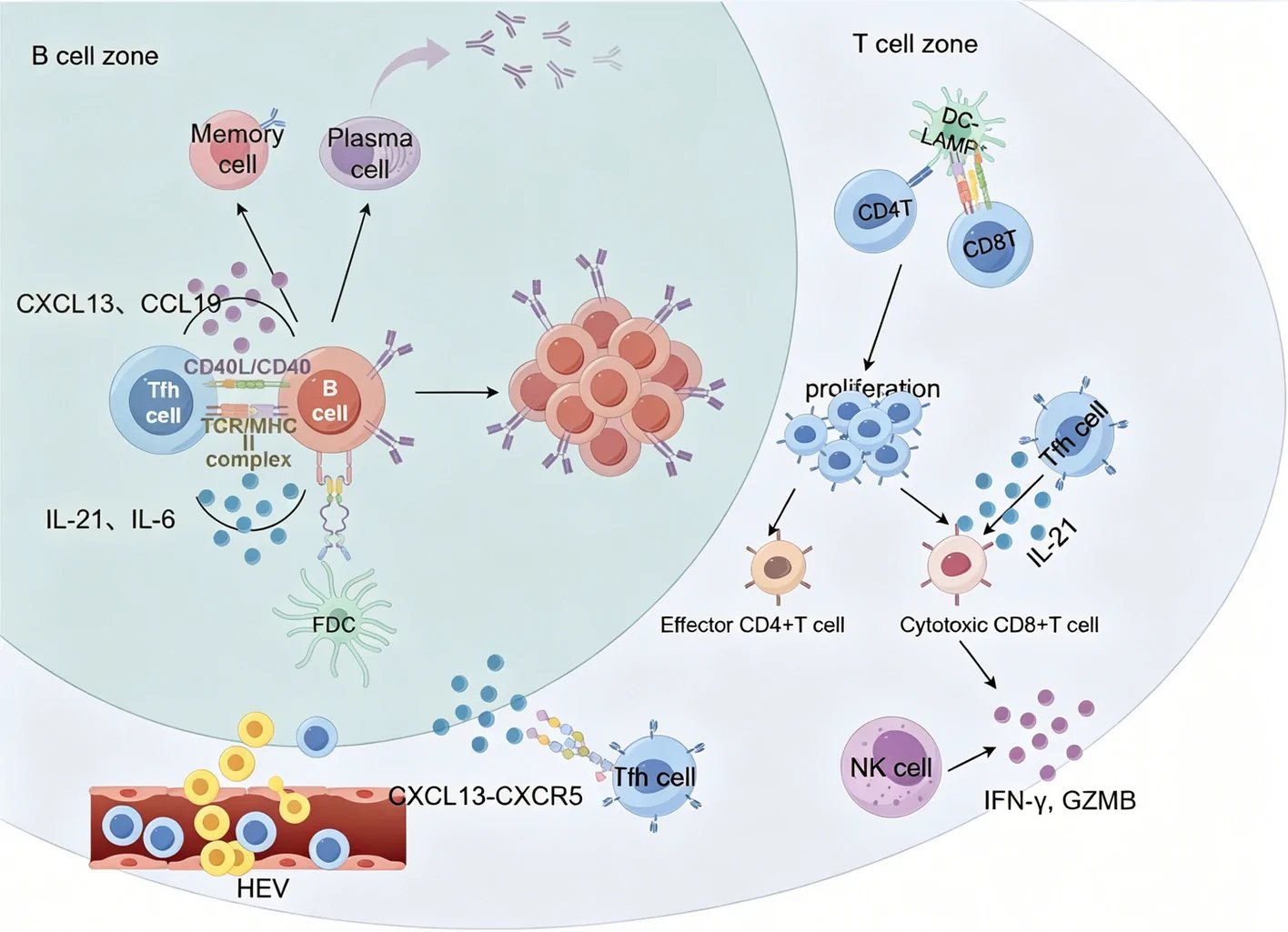
The mechanism of TLS in anti-tumor immunity. Fully mature TLS with intact germinal centers (GC), FDC networks and HEVs orchestrate coordinated cellular and humoral anti-tumor immunity. DC-mediated antigen presentation induces Tfh differentiation; Tfh cells support GC B-cell maturation to generate tumor-specific IgG antibodies, while boosting cytotoxic CD8⁺ T and Trm cell responses for durable tumor control. Created with figdraw.com

TLS serve as core immune mechanisms to antitumor in the tumor microenvironment (Fig. 4), replacing secondary lymphoid organs (SLO), by establishing in situ, structured, and highly timely local adaptive immune synapses. They are the primary mechanism driving anti-tumor responses [4]. Mature TLS (SFL-TLS) feature distinct T/B cell compartments, functional germinal centers (GC), and high endothelial venules (HEV), enabling direct antigen capture, presentation, and lymphocyte activation locally within the tumor without relying on draining lymph nodes to initiate a response [63, 142]. In the T-cell zone, DC‑LAMP + mature dendritic cells (DCs) efficiently present tumor antigens, forming stable immune synapses with CD4 + follicular helper T cells (Tfh) and CD8 + effector T cells, initiating antigen-specific clonal expansion [99, 117]; in the B-cell zone, CD21 +/CD35 + follicular dendritic cells (FDCs) retain antigen complexes for a long time, continuously driving B-cell receptor recognition and affinity maturation. Meanwhile, HEV efficiently recruits naive lymphocytes and memory cells into the TLS via the PNAd‑CD62L axis, continuously replenishing the immune cell pool and enhancing the efficiency of synapse formation [122, 143, 144]. This localized immune synapse significantly shortens the antigen presentation pathway and enhances the intensity of immune activation, serving as the structural basis and core initiating step for the anti-tumor effect of TLS.

### Effector functions orchestrated by TLS

TLS forms a multi-layered and robustly persistent anti-tumor effector functional network through the coordination of multi-lineage immune cells and the integration of humoral/cellular immunity, which is the core mechanism determining patient prognosis and immunotherapy response. At the cellular immunity level, activated CD8 + cytotoxic T cells, tissue-resident memory T cells (Trm), and NK cells within TLS acquire strong killing activity, directly infiltrate the tumor parenchyma, and release granzyme, perforin, and IFN‑γ; Tfh cells secrete IL‑21, further enhancing CD8 + T cell function and maintaining an immune memory phenotype [145, 146]. At the level of humoral immunity, B cells undergo somatic hypermutation (SHM) and class switch recombination (CSR) within the germinal center (GC), differentiate into IgG/IgA plasma cells, secrete tumor-specific antibodies, and mediate ADCC and CDC effects, directly killing tumor cells [93, 122]. In addition, tumor-associated lymphocyte (TAL) stimulator (TLS) constructs a concentration gradient through chemokines such as CXCL13 and CCL19/CCL21, continuously recruiting effector cells and maintaining an activated immune state, forming a complete closed loop of "activation-expansion-infiltration-killing", significantly enhancing the intensity and persistence of anti-tumor immunity [121, 147].

### Intercellular communication within the TLS niche

TLS relies heavily on a highly ordered, multidimensional intercellular communication network to facilitate dynamic interactions and functional regulation among immune cells. This network serves as a crucial molecular foundation for maintaining its maturity, stability, and antitumor activity. This communication system is composed of three types of signal coordination: Firstly, the chemokine and cytokine signaling axis, where CXCL13-CXCR5 regulates the recruitment and localization of Tfh and B cells, CCL19/CCL21-CCR7 regulates the migration of DCs and T cells, LTα1β2-LTβR drives the maturation of FDCs and the formation of HEVs, and IL-21, BAFF, and APRIL regulate B cell survival and differentiation [148–151]. Secondly, contact-dependent costimulatory/co-inhibitory signals, such as ICOS-ICOSL and CD40-CD40L, maintain immune activation, while PD-1-PD-L1 and CTLA-4 regulate the activation threshold and tolerance balance [79, 103]. Thirdly, the stromal cell support network, composed of fibroblasts (FRC) and antigen-presenting cancer-associated fibroblasts (apCAF), constructs a three-dimensional scaffold, secreting chemokines and adhesion molecules to coordinate the aggregation, compartmentalization, and spatial interaction of immune cells [24, 152].

## Preclinical advances and therapeutic implications

### Inducing TLS neogenesis: a novel therapeutic frontier

**Fig. 5:**
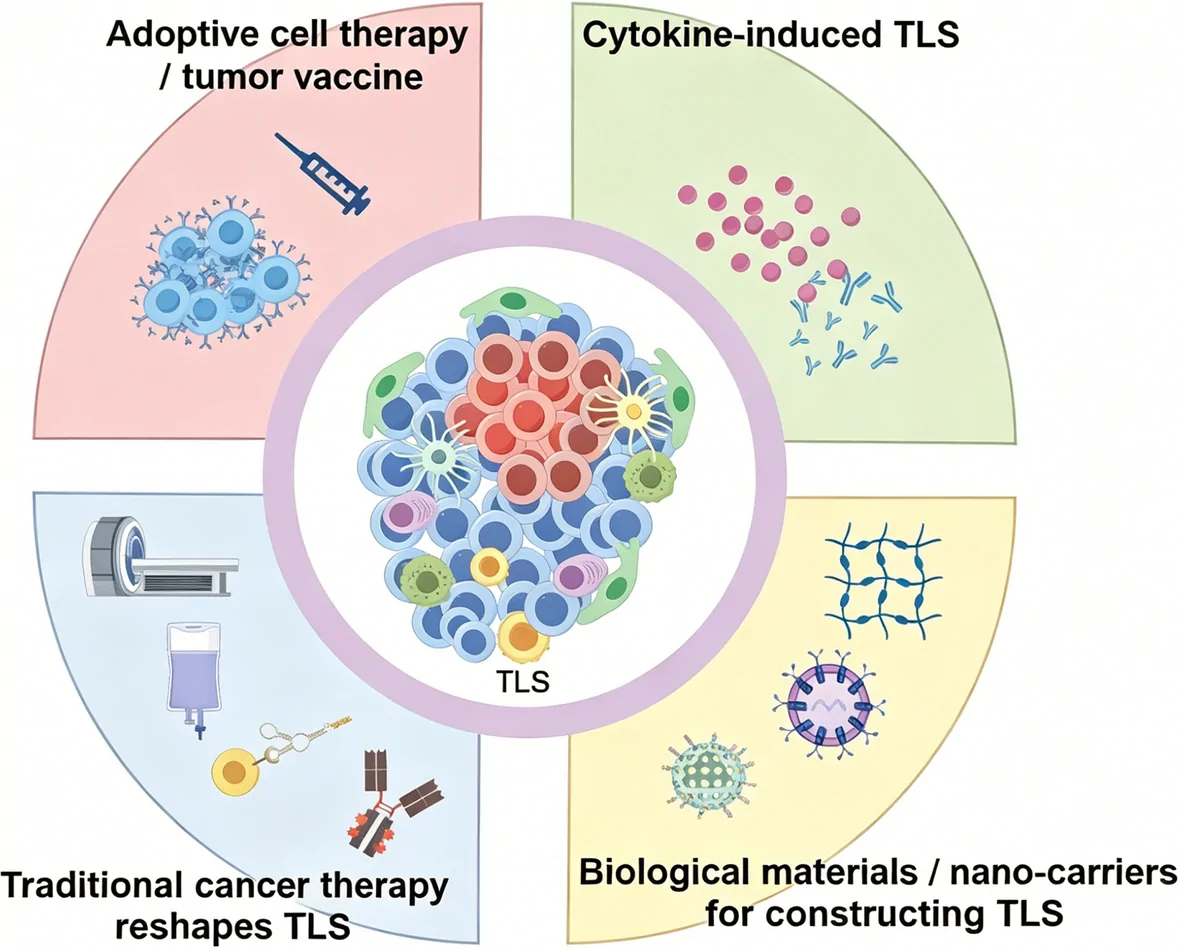
Therapeutic strategies targeting tertiary lymphoid structures (TLS) to reshape anti-tumor immunity. This schematic summarizes major approaches including chemokines, innate immune agonists, biomaterial scaffolds, gene delivery, and combination cancer therapies, which facilitate TLS neogenesis and boost antitumor immunity. Created with figdraw.com

Inducing the de novo generation and functional maturation of tertiary lymphoid structures (TLS) is a revolutionary therapeutic strategy for reshaping the tumor immune microenvironment and transforming "cold tumors" into "hot tumors" (Fig. 5). Its core lies in simulating the physiological lymphoid organ neogenesis program to establish an in situ immune center locally within the tumor, thereby reversing immune rejection, immune exhaustion, and treatment resistance [6, 15, 153]. This process is highly dependent on four core signaling axes: LTα1β2‑LTβR, LIGHT‑HVEM, CXCL13‑CXCR5, and CCL19/CCL21‑CCR7. By activating stromal cells to transform into lymphoid tissue organizer (LTo) cells, inducing the development of high endothelial venules (HEV), establishing clear T/B cell partitions and functional germinal centers, it reshapes the immune microenvironment from a structural level [154, 155]. Currently, a multi-dimensional and multi-pathway parallel induction system has been established: cytokine/chemokine targeted delivery is the most direct induction method. Local delivery of LIGHT, LTβR agonists, CXCL13, and CCL21 can efficiently promote HEV generation and TLS assembly, significantly enhancing the efficacy of immune checkpoint inhibitors (ICI) and overcoming drug resistance [40, 52, 156]. Natural immune agonists (STING, TLR3/4/9 agonists) trigger immunogenic cell death (ICD), activate dendritic cells and macrophages, simultaneously achieving antigen release and TLS neogenesis, forming a synergistic amplification effect of "antigen release—lymphatic neogenesis—immune activation" [157]. Biomaterials and three-dimensional scaffold systems provide a controllable local microenvironment for TLS induction. Biomaterials such as collagen sponges, hydrogels, cryogels, PLGA nanoparticles, and liposomes can achieve the controlled release and targeted delivery of chemotactic factors, cytokines, and immune activators, constructing a three-dimensional immune scaffold locally in the tumor area, directing the maturation of TLS, and enhancing its stability [158, 159]. This is particularly applicable to immune-desert tumors and postoperative residual lesions. Neoadjuvant immunotherapy/chemotherapy, oncolytic viruses, and targeted anti-angiogenic drugs can significantly enhance the quantity and maturity of endogenous tumor-associated lymphocytes (TLS) by improving vascular normalization and alleviating inhibitory signals. Among them, the PD-1/CTLA-4 combination therapy exhibits a significantly superior ability to induce TLS compared to monotherapy and is highly positively correlated with the rate of pathological complete response (pCR) [141, 160, 161]. More importantly, TLS induction possesses clear therapeutic selectivity, enhancing only anti-tumor immunity without enhancing the response to chemotherapy. Furthermore, the abundance of mature TLS can directly predict the degree of benefit from induction therapy, providing visual and quantitative indicators for achieving precise stratification [139].

### Harnessing TLS for enhanced adoptive cell therapies and vaccination

TLS, serving as the functional immune hubs in situ within tumors, systematically enhance the antitumor efficacy of adoptive cellular therapy (ACT) and therapeutic tumor vaccines, emerging as a pivotal synergistic strategy to overcome the bottleneck in solid tumor immunotherapy [1, 162]. In the context of tumor vaccines, the mature dendritic cells (DCs) and follicular dendritic cells (FDCs) within the tumor-loaded stimulator (TLS) efficiently capture, retain, and present vaccine antigens, continuously driving the activation of antigen-specific T cells and the humoral immune response of B cells [143, 163–165]. The TLS system, constructed from biomaterials such as hydrogels and cryogels, can simultaneously load vaccine antigens, DC cells, and LTβR agonists, forming functional lymphoid structures locally in the tumor, significantly enhancing the intensity of the anti-tumor immune response induced by the vaccine [166, 167]. TLS reshapes the immunosuppressive microenvironment, reduces inhibitory signals such as PD-L1 and TGF-β, and improves the tolerance of immune-desert tumors to ACT and vaccines; meanwhile, the effector cells reinfused by ACT and the immune response activated by vaccines can further promote the maturation and stability of endogenous TLS [52, 168]. Preclinical studies have confirmed that the hydrogel TLS system, carrying CAR-T and STING agonists, can significantly enhance local immune activation and tumor clearance at the resection site of pancreatic cancer, which is superior to simple intravenous infusion of CAR-T [168]. Artificial TLS loaded with antigens and chemokines can induce potent IgG humoral and cellular immunity, effectively inhibiting tumor growth and recurrence [163, 169]. In summary, the combined immune strategy centered around TLS, by constructing an in situ functional immune niche, provides a novel paradigm for overcoming the limitations of solid tumor treatment with adoptive cell therapy and tumor vaccines, holding broad prospects for clinical translation [162, 170].

### Challenges and caveats

Firstly, the heterogeneity and context-dependency of tumor-associated lymphocyte (TAL) function are severely underestimated. Immature TALs, peritumoral TALs, and inhibitory TALs enriched in Treg/Breg not only lack therapeutic value but may also promote tumor progression and immune evasion. Evidence of poor prognosis has been observed in liver cancer, breast cancer, and renal cancer [115, 129, 171]. Secondly, the precision of the induction strategy is insufficient. Systemic activation of pathways such as LTβR may trigger autoimmune reactions, lymphoproliferation, and systemic inflammation. Although local delivery is safer, biomaterials are prone to triggering foreign body reactions (FBR), leading to fibrous encapsulation and functional failure [172–174]. Thirdly, the manufacturing and regulatory challenges of clinical translation, TLS involves complex systems of biomaterials, recombinant proteins, and cellular components, which are costly and have unclear regulatory classification [175, 176]. Finally, TLS induction yields the best results in patients with intact immunity, tumor antigen positivity, and normal function of draining lymph nodes. However, patients with advanced disease, exposure to chemotherapy, immune senescence, or impaired lymphatic structures may not benefit from it [6, 141, 177]. Despite the challenges faced in this field, such as insufficient induction efficiency, difficulties in regulating maturity, foreign body reactions, systemic safety risks, and the absence of a standardized evaluation system, the immune microenvironment remodeling strategy centered on TLS neogenesis has demonstrated significant potential to transform immunosuppressive tumors into highly responsive ones. It has become an important frontier in the combination of immunotherapy, overcoming drug resistance, and enhancing long-term survival.

## Conclusions

TLS, serving as functional immune hubs in situ within tumors, exhibit dual functional roles determined by their maturity, spatial localization, and cellular composition. Mature TLS can significantly improve patient prognosis and enhance immunotherapy responses, whereas immature or inhibitory TLS may mediate immune evasion and poor prognosis. Targeting the induction of TLS neogenesis and functional maturation has emerged as a cutting-edge therapeutic strategy for reshaping the tumor immune microenvironment, enhancing the efficacy of adoptive cell therapy, and tumor vaccines, providing a novel direction and key targets for the next generation of precision immunotherapy.
